# MtDNA depleted PC3 cells exhibit Warburg effect and cancer stem cell features

**DOI:** 10.18632/oncotarget.9610

**Published:** 2016-05-26

**Authors:** Xiaoran Li, Yali Zhong, Jie Lu, Karol Axcrona, Lars Eide, Randi G. Syljuåsen, Qian Peng, Junbai Wang, Hongquan Zhang, Mariusz Adam Goscinski, Gunnar Kvalheim, Jahn M. Nesland, Zhenhe Suo

**Affiliations:** ^1^ Department of Pathology, The Norwegian Radium Hospital, Oslo University Hospital, Oslo, 0379, Norway; ^2^ Department of Pathology, Institute of Clinical Medicine, Faculty of Medicine, University of Oslo, Oslo, 0318, Norway; ^3^ Department of Oncology, The First Affiliated Hospital of Zhengzhou University, Zhengzhou University, Zhengzhou, Henan, 450052, China; ^4^ Department of Epidemiology and Biostatistics, College of Public Health, Zhengzhou University, Zhengzhou, Henan, 450001, China; ^5^ Department of Medical Biochemistry, University of Oslo and Oslo University Hospital, Oslo, 0372, Norway; ^6^ Department of Urology, The Akershus University Hospital, Lørenskog, 1478, Norway; ^7^ Department of Radiation Biology, Institute for Cancer Research, The Norwegian Radium Hospital, Oslo University Hospital, Oslo, 0379, Norway; ^8^ Laboratory of Molecular Cell Biology and Tumor Biology, Department of Anatomy, Histology and Embryology, Peking University Health Science Center, Beijing, 100191, China; ^9^ Department of Surgery, The Norwegian Radium Hospital, Oslo University Hospital, Institute for Clinical Medicine, Faculty of Medicine, University of Oslo, Oslo, 0379, Norway; ^10^ Department of Cell Therapy, Cancer Institute, The Norwegian Radium Hospital, Oslo University Hospital, Oslo, 0379, Norway

**Keywords:** mitochondrial DNA, Warburg effect, hypoxia, transcriptome analysis, cancer stem cells

## Abstract

Reducing mtDNA content was considered as a critical step in the metabolism restructuring for cell stemness restoration and further neoplastic development. However, the connections between mtDNA depletion and metabolism reprograming-based cancer cell stemness in prostate cancers are still lack of studies. Here, we demonstrated that human CRPC cell line PC3 tolerated high concentration of the mtDNA replication inhibitor ethidium bromide (EtBr) and the mtDNA depletion triggered a universal metabolic remodeling process. Failure in completing that process caused lethal consequences. The mtDNA depleted (MtDP) PC3 cells could be steadily maintained in the special medium in slow cycling status. The MtDP PC3 cells contained immature mitochondria and exhibited Warburg effect. Furthermore, the MtDP PC3 cells were resistant to therapeutic treatments and contained greater cancer stem cell-like subpopulations: CD44+, ABCG2+, side-population and ALDH^bright^. In conclusion, these results highlight the association of mtDNA content, mitochondrial function and cancer cell stemness features.

## INTRODUCTION

Prostate cancer (PCa) is the second leading cause of cancer death among men in Western countries. In the year 2014, 220,800 new PCa cases were diagnosed in United States and 29,480 PCa patients died of the disease [1]. PCa usually begins with androgen-dependent growth with positive androgen receptor (AR+) expression, showing sensitive to androgen deprivation therapy (ADT) [2, 3]. Androgen deprivation therapy (ADT) is a common treatment for patients diagnosed with prostate cancer and this treatment can be accompanied by surgery (bilateral orchiectomy) or medical castration with luteinizing hormone-releasing hormone (LHRH) agonists. ADT with anti-androgens remains as the main treatment for later stage PCa. Although ADT is initially effective, androgen independent prostate cancer relapse usually occurs (castration resistant prostate cancer (CRPC)) [4]. Most of CRPCs show aberrant androgen receptor expression and are frequently clinically aggressive [3, 5]. CRPC patients frequently develop metastases and therapeutic resistant properties, the main reason for treatment failure of prostate cancer patients [6–9].

It has been known for decades that mitochondrial DNA (mtDNA) plays important roles in carcinogenesis [10–12]. It has been reported that mtDNA depletion may result in androgen-independent growth and a CRPC phenotype when mtDNA replication is inhibited in the AR+ prostate cancer cells [13]. However, the phenomenon has not been studied in the AR- CRPC cell line PC3. The PC3 cell line was established in 1979 from an AR- prostate cancer bone metastasis in a clinical stage IV 62-years-old Caucasian male patient [14]. PC3 cells are of the following features: (1) AR- with reduced response to androgens, epidermal or fibroblast growth factors; (2) high metastatic potential; (3) significantly enhanced therapeutic resistance [15]. Until now no mtDNA depletion report using this aggressive CRPC cell line is available in literature.

In addition to conduct oxidative respiration, mitochondria are also involved in other vital cellular functions such as bio-molecule synthesis, apoptosis and proliferation. Recently, the importance of oxidative respiratory stress in cell stemness restoration during somatic cell reprograming process in induced pluripotent stem cells (iPSCs) has been documented [16]. Synchronized with stemness restoration during the induction of iPSCs, initially matured mitochondria in the somatic cells gradually show naïve/immature status along with reduced mtDNA content and decreased oxidative phosphorylation (OXPHOS) function and highly glycolytic metabolism dependence [17–19]. Thus iPSC and cancer stem cell studies indicate that cancer cell stemness may be associated with glycolysis, a well-known feature of Warburg effect [20].

Otto Heinrich Warburg was the first to demonstrate cancer cells carrying out a modified glucose metabolism. Instead of using the oxidative pathway ending up with CO_2_ and water, sharply increased proportion of glucose molecules in cancer cells was converted into lactate and excluded from the cell, even where the oxygen was sufficient [21]. He concluded that the division of labor in energy production (ATP) even in the most rapidly growing tumor cells usually does not exceed 50% for the glycolytic contribution [22]. Besides the cancer cell “fermentation” feature, i.e., aerobic glycolysis, the Warburg effect is now considered to be more than just a unique energy metabolism program. The Warburg effect represents a steadily modified metabolic state, allowing energy substrates, particularly glucose, to shuttle into variable biomass synthesis pathways. Facilitated by the sufficient reducing force (mainly NADPH) bypass generated, the incomplete oxidized glucose molecules are utilized to synthesize biomaterials such as lipids and amino acids [23]. Thus, benefiting from “Warburg program”, these abundant biomass molecules are subjected to build up new cells, especially for stem cells and cancer cells to meet their proliferation requirements. It has been noticed for years that in the AR- prostate cancers, the mtDNA depletion protects cells from anoikis and gives a cell metastasis potential, which might be powered by metabolic reprogramming towards Warburg effect. Therefore, establishing mtDNA depletion (MtDP) cancer cell line with CRPC features may provide ideal model for further studies of MtDP, Warburg effect and cell stemness.

## RESULTS

### The MtDP PC3 cells are derived from and adapted for long-term EtBr treatment

**Figure 1:**
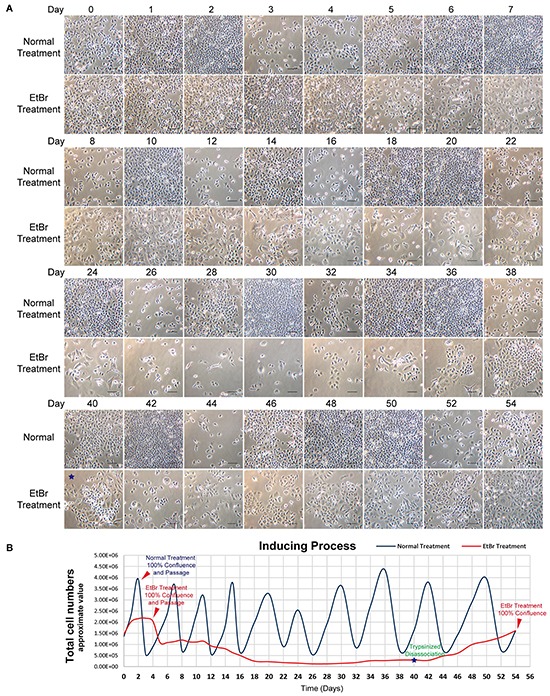
Long-term EtBr treated cells are adapted MtDP cells **A.** Representative series photographs of typical 54 days inducing process are shown. Normal treatment means PC3 cells were cultivated in normal medium. EtBr treatment means PC3 cells were cultivated in the medium containing 2μg/ml of EtBr, 1mM sodium pyruvate and 50μg/ml uridine. Cell growth status in normal medium is displayed as reference. **B.** Curves of cell numbers counted for both cells. The red arrowheads denote confluence of the cells. Blue line represents PC3 cells treated in normal medium while red line represents the PC3 cells treated in the medium containing EtBr, pyruvate and uridine. Each blue line peak indicates a passaging. For the PC3 cells treated in the medium containing EtBr, pyruvate and uridine (red line), only one passaging was performed on the 4th day. Scale bar = 100μm.

To obtain MtDP PC3 cells, we utilized a classic method by using EtBr with sufficient extra sodium pyruvate plus uridine as supplements [24]. Based on our observations, a typical inducing period of MtDP PC3 cells usually lasted 52~60 days, depending on the extents of optimizing supplemental gradients and vessel type. As shown in Figure 1A, the whole inducing process could be considered in 3 different stages. In the first 4~5 days the PC3 cells exhibited sharply reduced proliferative activity with morphological alternations. Nevertheless, the EtBr treated cells still could manage to reach 100% confluence. However, after the cells were passaged at ratio 1:2, then the second inducing stage began, normally for a month. During this period, up to 90% of the PC3 cells gradually showed adaptation failure to the EtBr treatment and detached from the flask. Majority of the detached cells were confirmed dead and were excluded from the daily medium change. Finally, only ~10% of the cells survived the treatment and thus initiated stable cell colonies. Interestingly, in this period a large number of the survived cells were also found with features of epithelial-mesenchymal transition (EMT), and our real-time video also suggested that the cells started to show greater mobility (data not shown). The third period usually took another 20 days, showing slow proliferation to reach confluence. Furthermore, we estimated the approximate cell numbers by daily captured images to draw a consecutive monitoring chart. In Figure 1B, the blue line peaks denote the cell passaging for wild type PC3 cells, which usually performed every 3~4 days. However, the EtBr treated cells during the typical two-month induction process were passaged only once, which happened at day 4. Since only few colonies were developed in this stage, and the cells in these colonies might experience cell contact-induced growth inhibition, an additional trypsinized dissociation was performed at day 40 for optimization, without splitting the flask. As shown in Figure 1A and 1B, the lowest number of cells in the induction process was observed at day 26~28. The cells exhibiting faster growth were observed around day 42, and reached 100% confluence at day 54.

### The MtDP PC3 cells are mtDNA depletion, pyruvate and uridine auxotrophic and slow cycling

**Figure 2:**
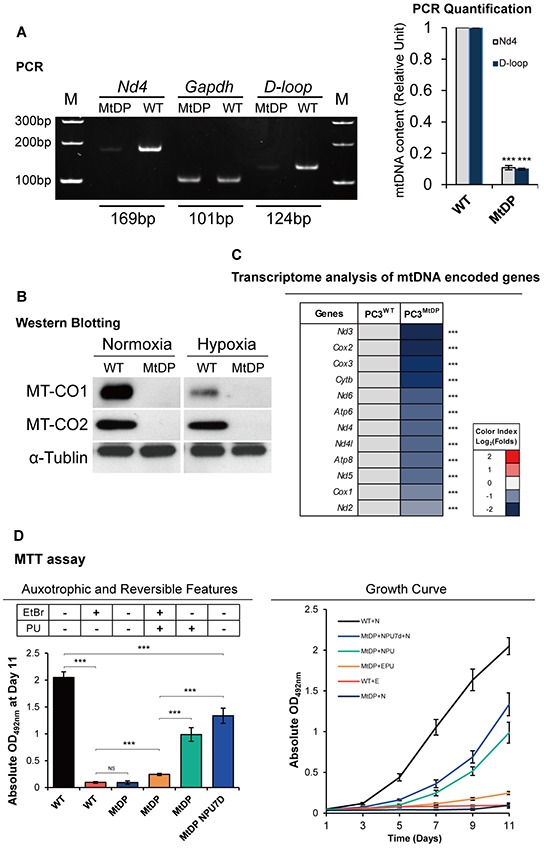
The MtDP PC3 cells are mtDNA depletion, pyruvate and uridine auxotrophic and slow cycling **A.** Representative PCR result of the *Nd4* and *D-loop* mtDNA sequences with nuclear encoded gene *Gapdh* as control is shown on the left, and the amplification of mtDNA regions in the MtDP PC3 cells relative to the WT PC3 cells was calculated upon normalization to the reference *Gapdh* (right). The data are presented as means ± S.D (n=3). **B.** Protein levels of mtDNA encoded MT-CO1 and MT-CO2 were evaluated by western blotting of total protein extracts using anti MT-CO1 and MT-CO2 antibodies, α-tubulin signal was used as loading control. **C.** Transcriptome analysis of mitochondrial encoded genes in WT and MtDP PC3 cells. A color-coded index bar indicates the ratios. (FPKM value, Log_2_ (MtDP/WT)). **D.** MTT assay results. WT and MtDP PC3 cells were maintained in variable cell culture media with different gradients of EtBr and P (pyruvate) and U (uridine) presence (+) or not (−). The abbreviations are of followings: N: EtBr-/PU-; NPU: EtBr-/PU+; E: EtBr+/PU-; EPU: EtBr+/PU+. MtDP+NPU7d+N means MtDP PC3 cells were pretreated in NPU medium for 7 days before MTT assay, and then maintained in N medium in the experiment. The data are presented as means ± S.D (n=3). Statistical significance: *p<0.05, **p<0.01, ***p<0.001.

To confirm MtDP [25], we measured relative mtDNA content by PCR with specific primers targeting different mtDNA regions. As shown in Figure 2A, EtBr treatment resulted in more than 85% reduction of the PCR products of *Nd4* and *D-loop*. Western blotting analysis with mtDNA encoded cytochrome oxidase I (MT-CO1) and cytochrome oxidase II (MT-CO2) confirmed that MT-CO1 and MT-CO2 protein levels in long term EtBr treated PC3 cells were undetectable (Figure 2B). Control cells showed abundant protein products.

To verify the blotting, we treated both cells with hypoxic cultivation for 48h, since it has been proved that oxygen tension regulates the expression of mtDNA encoded complex I and IV genes [26]. The results showed that wild type (WT) PC3 cells were highly sensitive to low oxygen (1.5% O_2_) and exhibited different degrees of MT-CO1/CO2 protein expression reduction. However, the protein expression in the mtDNA depletion PC3 cells (MtDP PC3 cells) remained undetectable. Transcriptome analysis (Figure 2C) confirmed that mtDNA encoded gene expressions were sharply reduced in the long-term EtBr treatment group. Together, these results confirmed the successful establishment of MtDP PC3 cells. Then we explored cell proliferation and auxotrophic properties by using MTT assay in consideration of the effect of EtBr and pyruvate plus uridine (PU). As shown in Figure 2D on the left, neither WT PC3 cells survive in the PU free normal medium with EtBr nor the MtDP cells survive in the EtBr free normal medium without PU. But MtDP PC3 cells survive in the medium with EtBr and PU in slow proliferation rate. If the MtDP cells were cultivated in normal medium with PU, about ~50% proliferation rate recovery could be seen at day 11 according to the MTT experiment, compared to the PC3 WT cells in normal medium. However, if the MtDP cells were pretreated in normal medium with PU, but without EtBr for 7 days before MTT assay (MtDP NPU7D), significantly more cells were proliferative, indicating the auxotrophic to PU is reversible in the PC3 MtDP cells. As shown in Figure 2D on the right, MtDP cells are PU auxotrophic and EtBr in the medium keeps the MtDP cells in slow cycling with the presence of PU, and withdrawing EtBr from the medium significantly reverses the auxotrophic for PU, and recovers cell proliferative capacity.

### MtDP PC3 cells contain immature mitochondria

**Figure 3:**
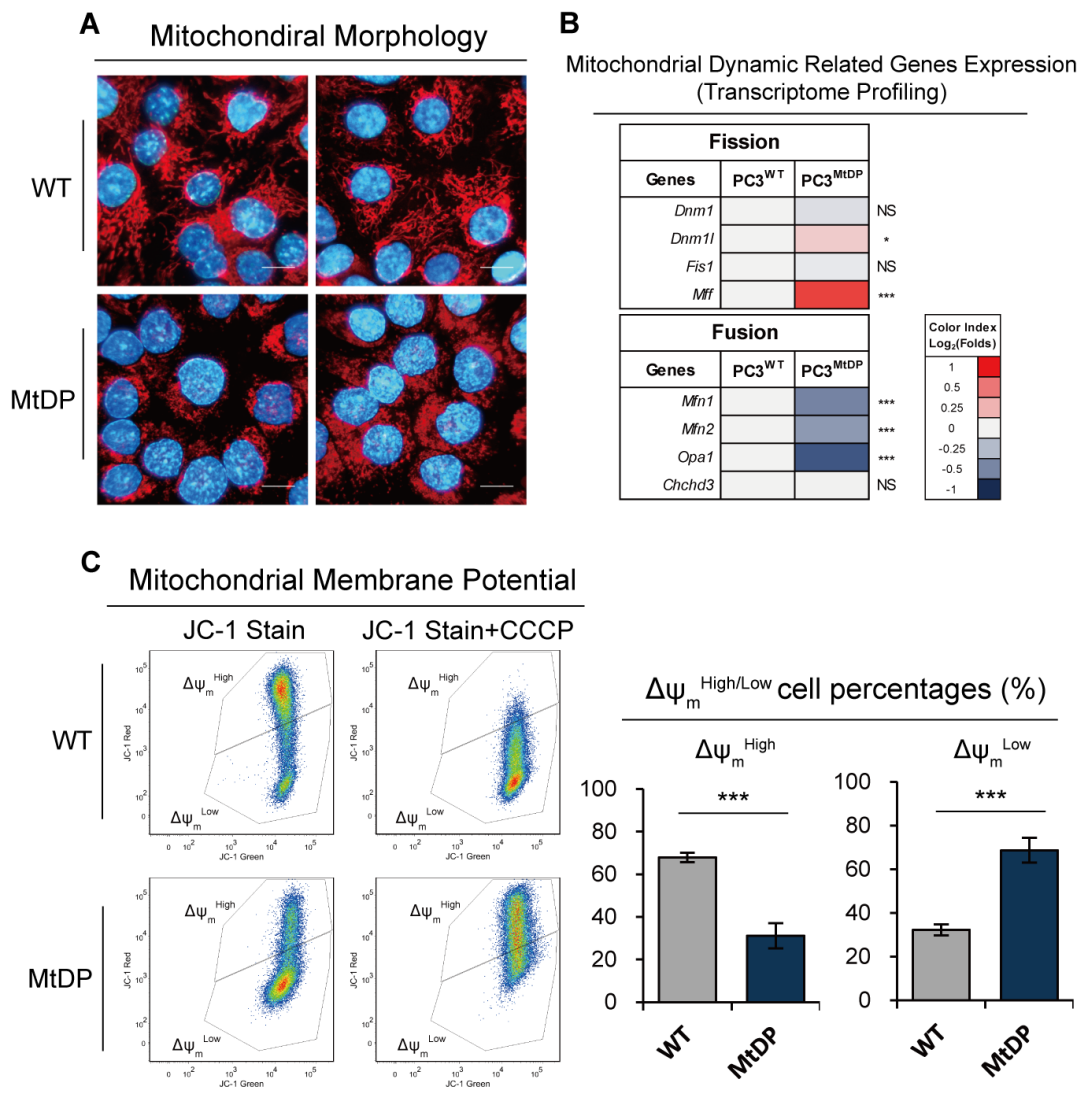
MtDP PC3 cells show immature mitochondria **A.** WT and MtDP PC3 cells were stained with MitoTracker® Red FM (mitochondria, red) and Hoechst33342 (nuclei, blue). Representative two images from independent tests are displayed for WT and MtDP PC3. Scale bar= 20μm. **B.** Transcriptome analysis of mitochondrial dynamic related genes in WT and MtDP PC3 cells. A color-coded index bar indicates the ratios. (FPKM value, Log_2_ (MtDP/WT)). **C.** Evaluation of Δψ_m_ in WT and MtDP PC3 cells determined by Δψ_m_-sensitive JC-1 dye staining and flow cytometry. Pretreatment with 2μM CCCP was used as staining control. The percentages of cells with capacity to form JC-1 aggregates (Δψ_m_^High^) and JC-1 monomer (Δψ_m_^Low^) were determined in each group (the histograms). The data are presented as means ± S.D (n=3).

As shown in Figure 3A, mitochondrial morphology was visualized by probe MitoTracker RED FM™ (red). Cell nuclei were stained by Hoechst 33342 (blue). Tubule branch-like mitochondria were found widely distributed in the cytoplasm in the wild type PC3 cells. Mitochondria in the MtDP PC3 cells were highly fragmented, dot-like and often had perinuclear localization. Since mitochondria are highly dynamic organelles with constant fission and fusion balance, we next investigated the expression of mitochondrial dynamic related genes by transcriptome profiling. As shown in Figure 3B, besides the *Dnm1l* and *Mff* gene expression up-regulation, no gene expression contributing to mitochondrial fission is down-regulated in the MtDP cells. However, 3/4 of the fusion regulatory genes were suppressed in the MtDP cells compared with the wild type PC3 cells. To further explore mitochondrial function alteration, we next measured mitochondrial membrane potential (Δψ_m_) by using mitochondrial Δψ_m_ sensitive probe JC-1. According to the experiment principle, mitochondrial depolarization is indicated by a decrease in the red/green fluorescence events ratio, and the potential-sensitive color shift is due to concentration-dependent formation of red fluorescent JC1-aggregates. As shown in Figure 3C, on the left, WT PC3 cells possess rather well-maintained Δψ_m_ and exhibit high capacity to form JC-1 aggregates (65~70% in proportion) whereas MtDP PC3 cells show a significantly reduced proportion (26~37%). On the contrary, MtDP PC3 cells exhibit abundant cells with collapsed Δψ_m_ (62~73% Δψ_m_^Low^), while only around 29~35% WT PC3 contain cells with Δψ_m_^Low^. For the positive controls, sufficient carbonyl cyanide 3-chlorophenylhydrazone (CCCP) was added to pretreat the cells at a final concentration of 2μM. As shown in Figure 3C, WT PC3 cells are sensitive to the 2μM uncouple regent CCCP and exhibit collapsed Δψ_m_ after the treatment. However MtDP PC3 cells are shown with aberrant results.

Theoretically, the absence of mtDNA-encoded proteins should directly lead to mitochondrial function defect. For basic mitochondrial functions, Δψ_m_ is regarded as the fundamental driving force to conduct OXPHOS and generate ATPs. The collapsed Δψ_m_ in the MtDP cells suggests the possibility that the cells with mitochondrial oxidation defects may survive with alternative metabolic programs. Hence, we next evaluated glycolysis and OXPHOS efficiency in both WT and MtDP PC3 cells.

### MtDP PC3 cells exhibit Warburg effect with significantly reduced ATP production

**Figure 4:**
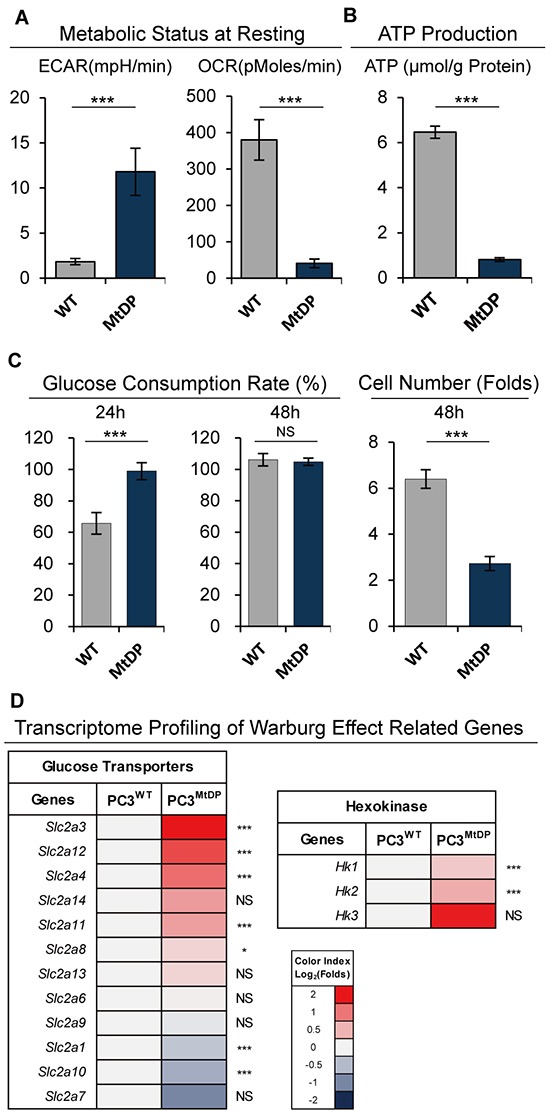
MtDP PC3 cells exhibit Warburg effect **A.** ECAR and OCR values measured under resting circumstance in WT and MtDP PC3 cells. The data are presented as mean ± S.D (n=3). **B.** The ATP content in WT and MtDP PC3 cells was determined by using centrifuged supernatant of total cell lysis. The measured ATP concentration was normalized by total protein concentration in each sample. The data are presented as means ± S.D (n=4). **C.** Glucose concentration was determined in cell culture medium of each group as described in material and methods. Fresh medium was used as control for glucose consumption ratio calculation. The data are presented as means ± S.D (n=3). The cell numbers were counted and amplification folds are showing on the right (final/start). **D.** Transcriptome analysis of hexokinase and glucose transporter family genes in WT and MtDP PC3 cells. A color-coded index bar indicates the ratio. (FPKM value, Log_2_ (MtDP/WT)). Statistical significance: *p<0.05, **p<0.01, ***p<0.001.

To evaluate glycolysis reprogramming and OXPHOS, we measured the oxygen consuming rate (OCR) and extracellular acidification rate (ECAR) in the WT and MtDP PC3 cells with a Seahorse extracellular flux analyzer through a mito-stress assay. As shown in Figure 4A, WT PC3 cells were relatively OXPHOS active, showing relatively high oxygen consumption rate but limited acidification rate (OCR ~380 pMoles/min and ECAR ~2 mpH/min). On the contrary, MtDP PC3 cells show 10-fold reduced oxygen consumption with a 6-fold higher acidification rate (OCR ~40 pMoles/min and ECAR ~12 mpH/min). Meanwhile, MtDP PC3 cells did not respond to all of the regents present in the mitostress assay (Supplementary Figure S1A), thereby demonstrating alternative a rather electron transport chain (ETC)-independent respiration in the MtDP cells.

We then measured intracellular ATP contents to evaluate the effect on the ETC defect. As shown in Figure 4B, ATP level in the MtDP PC3 cells is ~6.5 fold lower than that in the WT PC3 cells (<1μmol/g protein in the PC3 MtDP vs ~6.5μmol/g protein in the PC3 WT). Collectively, the above results confirm that the MtDP PC3 cells contain dysfunctional mitochondria and exhibit highly glycolytic feature. Considering the possibility that the high ECAR value of the MtDP PC3 cells potentially indicates the Warburg effect [27, 28], we next examined the glucose uptake capacity in both cell types. As shown in Figure 4C, MtDP PC3 cells have around two folds faster glucose uptake at 24h. These results reveal that the glucose in the medium was consumed up by the MtDP PC3 cells at 24h culture (~99%) while the WT PC3 only used ~64% of the glucose in the medium. At 48h culture, the glucose in the medium for the wild type PC3 cells was also exhausted (~100%). In addition, since the experiment was only for estimating glucose consumption in a proliferating cell population, the uneven cell numbers caused by different division ratios after 48h cultivation were not normalized.

Since the above results support the suggestion that MtDP PC3 cells are typical Warburg effect-linked, we next asked how Warburg effect related genes changed in these cells according to recent publications. As shown in Figure 4D, gene expression in 2/3 of the hexokinase family members was upregulated in the MtDP cells, including: *Hk1* and *Hk2*. Furthermore, specific glucose transporters were found upregulated as well, such as: *Slc2a3*, *Slc2a12* and *Slc2a14*. Particularly, there was 50-fold higher *Slc2a3* mRNA expression in the MtDP PC3 cells compared to the WT PC3 cells (FPKM value). Further transcriptome analysis with main glucose metabolism pathways are shown in the Supplementary Figure S1B and S1C.

Taken together, the results did suggest a significant Warburg effect in the MtDP PC3 cells. Since the high glycolytic metabolism has been associated with cell stemness, we next explored the influence on cell stemness in the MtDP PC3 cells.

### The CD44^+^/Δψ_m_^Low^ is the main subtype in the PC3 MtDP cells

**Figure 5:**
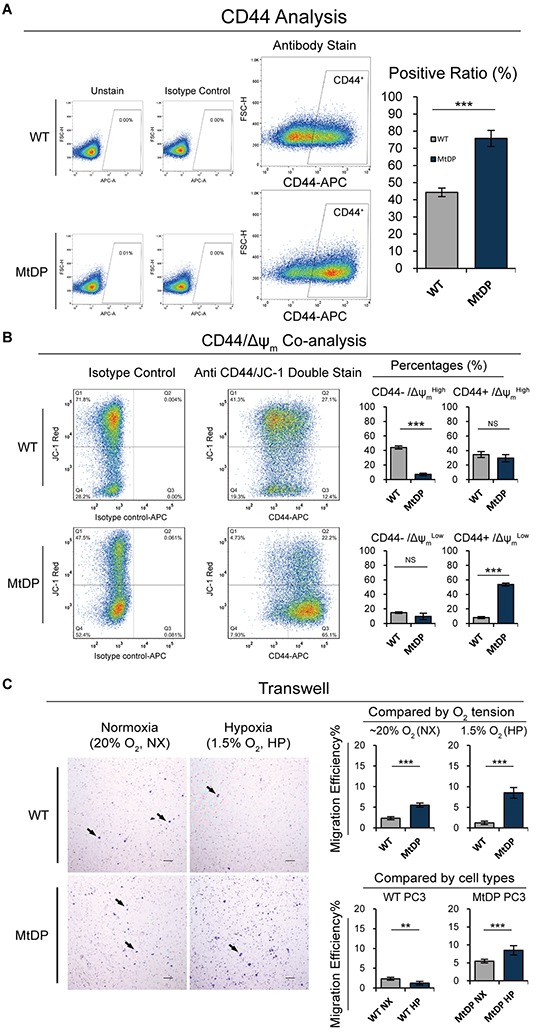
The flowcytometric CD44, CD44/Δψm co-analyses and transwell experiment results **A.** The CD44 expression level was assessed by flow cytometry for each group. The WT and MtDP PC3 cells were stained with a fluorophores conjunct CD44 antibody. The unstained and isotype control staining samples were used to confirm positive signal (left). Data are plotted in the right histogram, and data are presented as means ± S.D (n=3). **B.** Δψ_m_ and CD44 expression levels were co-analyzed by flow cytometry. The WT and MtDP PC3 cells were double stained with Δψ_m_-sensitive JC-1 dye (JC-1 aggregates, axis Y) and fluorophore conjuncted CD44 antibody (axis X). The staining of isotype control antibody was used to confirm CD44 positive events and the JC-1 staining was used to verify the Δψ_m_ condition in cells (left). The data of four different combinations with CD44^+/−^ and Δψ_m_^High/Low^ are plotted on the right histograms. Data are expressed as mean ± S.D (n=5). **C.** Cell migration capacity under normoxic and hypoxic circumstances were assessed by transwell assay. Black arrows point to the cells penetrated through polycarbonate membrane (left). The migrated cells were counted and the data are displayed in charts on the right, and the data present as mean ± S.D (n=3). Scale bar = 100μm. Statistical significance: *p<0.05, **p<0.01, ***p<0.001.

Since the cell adhesion molecule CD44 has been linked in different cancer stem cells such as breast, liver, ovarian, pancreatic and prostate cancers [29], we next evaluated CD44 expression status by flow cytometry. As shown in Figure 5A, a significantly increased CD44 reactivity is revealed in the MtDP PC3 cells (~45% positive in WT vs over 70% positive in MtDP cells). In the context of collapsed Δψ_m_ (JC-1 staining) in the MtDP cells, we simultaneously detected CD44 and mitochondrial Δψ_m_ by flow cytometry. As shown in Figure 5B, 54% of the MtDP PC3 cells are CD44 positive with depolarized mitochondrial membrane potential (CD44^+^/Δψ_m_^Low^) while there are only up to 10% of such cells in the WT PC3 cells. The WT PC3 cells show mainly CD44^−^/Δψ_m_^High^ phenotype (~7% in the MtDP PC3 vs ~45% in the WT PC3 cells, respectively).

### MtDP PC3 cells exhibit greater metastatic potential

Metastasis is responsible for as much as 90% of cancer-associated mortality [30]. However, recent studies have revealed that most critical tumor metastatic mechanisms are highly related with vicious cancer stem cells features. For example, CSCs have higher capacity to degrade extracellular cellular matrix (ECM) and exhibit potential homing features. To ask whether the MtDP cells exhibited greater metastasis capability, we performed transwell migration assay. For the experiment, human granulocyte colony stimulating factor (G-CSF) was applied to simulate artificial chemotactic resource. In addition, since hypoxia was found to facilitate MtDP cells to reduce the ROS level (data not shown), which might provide advantages for MtDP cell growth, hypoxia treatment was also applied in this assay. As shown in Figure 5C, under normoxic condition (~20% oxygen, NX) MtDP PC3 cells show more cells penetrating through 8.0μm diameter pore transwell membrane compared to the WT PC3 cells (~2% of WT vs ~5.5% of MtDP). However, hypoxic cultivation (HP) displayed distinct effect on WT and MtDP PC3 cells: increased penetration efficiency in the MtDP PC3 cells but decreased penetration efficiency in the WT PC3 cells (WT NX ~2% vs WT HP ~1%; MtDP NX ~5.5% vs MtDP HP ~8.5%, respectively).

The results suggest that PC3 MtDP cells have higher potential to develop metastasis under *in vivo* conditions where OXPHOS stress exists, since hypoxic circumstances may positively enhance the performance of MtDP PC3 cells with CSCs feature. However, the temporal hypoxic circumstance has negative influence on the WT PC3 cells.

### MtDP PC3 cells exhibit greater therapeutic resistance

**Figure 6:**
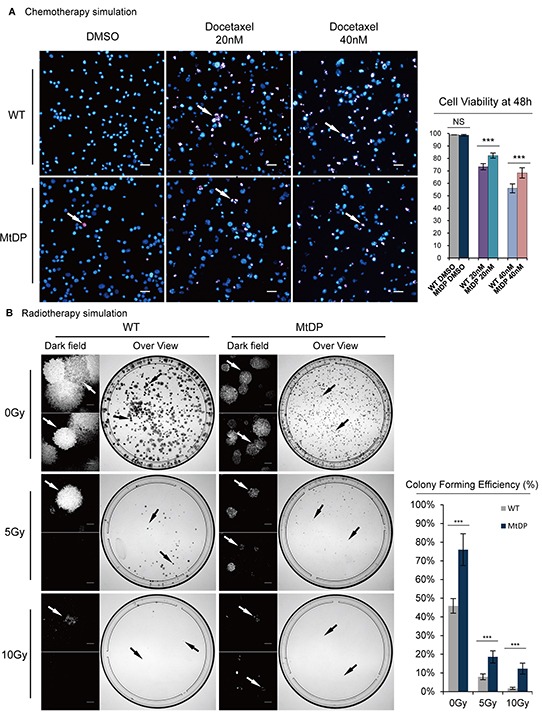
PC3 MtDP cells exhibit therapeutic resistance **A.** Both WT and MtDP PC3 cells were treated with 0nM (untreated, with equal volume DMSO), 20nM and 40nM docetaxel for 48h, respectively, and then the cells were stained with 5μg/ml PI and Hoechst33342 and visualized under fluorescent microscopy. The viable cells are only shown in blue (normal nuclei morphology) and dead cells are shown in a range of pink to white color (fragmented nucleus). Arrows point to representative dead/apoptotic cells. Scale bar = 50μm. Cell viabilities are showing in the histograms on the right. Data are presented by mean ± S.D (n=3). **B.** Representative images of irradiation examinations are showing on the left. 0Gy, 5Gy and 10Gy X-ray irradiations were given to WT and MtDP PC3 cells respectively, before the cells were incubated for modified colony formation assay for 16 days. Then, the cells were fixed and stained with 0.1% (w/v) crystal violet. The cell colonies were counted in a G: BOX multifunction imaging instrument (indicate by black arrow). For those dishes where the cells were irradiated at 5Gy and 10Gy, cell or small colony counting was performed under a dark field microscopy. Scale bar = 300μm. Showing on the right, histogram shows colonies/singular cells counted for both groups. The data are presented as means ± S.D (n=3). Statistical significance: *p<0.05, **p<0.01, ***p<0.001.

Based on the previous studies, we next asked whether the MtDP cells were more resistant to clinical treatments. As shown in Figure 6A, after 48h treatment of 20nM and 40nM docetaxel respectively, significantly more WT PC3 cells show positive staining with propidium iodide (PI) and verified dead. In summary, we found ~9% more MtDP cells survived 20nM docetaxel treatment compared with WT PC3. However, when the cells were treated with 40nM docetaxel, even more MtDP cells survive the treatment compared to the WT PC3 cells (~12% more MtDP cells survived). The results suggest that MtDP PC3 cells are less sensitivity to docetaxel treatment.

To evaluate radiotherapy response, we next performed a modified colony formation assay after subjecting cells to different doses of X-ray irradiation. To accomplish this, we seeded the cells into 100mm dishes and allowed the cells attach to vessel overnight before X-ray irradiation. As shown in Figure 6B, on the top, without X-ray irradiation, MtDP PC3 cells show significantly more small colonies than WT PC3 cells, in which fewer but larger colonies are present (~30% more colonies in the PC3 MtDP cells formed compared with WT). However, after either 5Gy or 10Gy X-ray irradiation, size and number of the colonies of both groups were reduced. Interestingly, microscopy examination could clearly see the survival difference (dark field). Around ~8% of the WT PC3 cells and 18% of the MtDP cells survived 5Gy irradiation, respectively. After 10Gy irradiation, only ~1.7% of the WT PC3 cells, but ~12% of the MtDP cells survived, respectively.

### MtDP PC3 cells exhibit up-regulation of CSC markers

**Figure 7:**
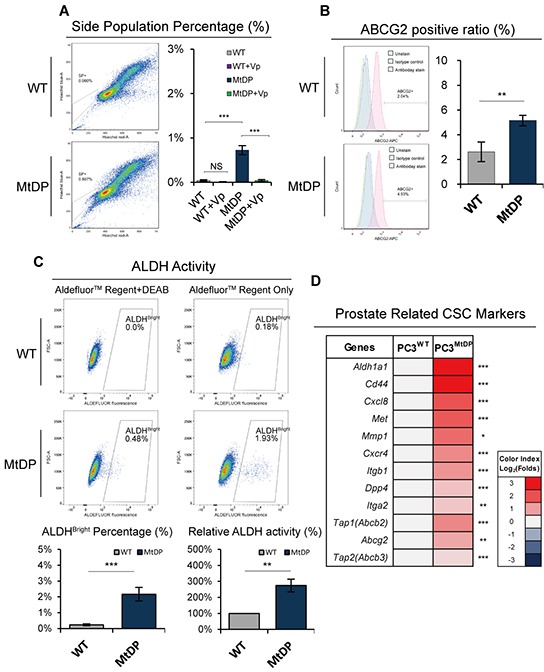
MtDP PC3 cells exhibit enhanced cancer stemness features **A.** The SP cells in WT and MtDP PC3 cells were identified by flow cytometry with the Hoechst33342 based staining procedure. The SP cells displayed in plots show a tail-like sub-population close to the G0/G1 phase (on the left). Error bars stand for mean ± S.D (n=4) (on the right). **B.** ABCG2 expression level in WT and MtDP PC3 cells was assessed by flow cytometry with fluorophore conjuncted ABCG2 antibody. Representative flow cytometry figures are shown on the left. Histogram of the ABCG2 expression is shown on the right. The data are presented as means ± S.D (n=3). **C.** ALDH enzyme activity of WT and MtDP PC3 cells were evaluated by flow cytometry with a commercial ALDEFLUOR™ kit as described in material and methods. Represent images are shown on the upper part. The ALDH^bright^ cell proportions and relative ALDH activity (fluorescent intensity) are shown in lower part as histograms. Four independent experiments were carried out. Error bars stand for mean ± S.D (n=3). **D.** Transcriptome analysis of prostate cancer stem cell-related genes in WT and MtDP PC3 cells. The ratios are indicated by color-coded index bars. (FPKM value, Log_2_ (MtDP/WT)). Statistical significance: *p<0.05, **p<0.01, ***p<0.001.

Since the side-population (SP) and ABCG2 expression have been linked to chemotherapeutic resistance [31, 32], we next examined both factors by flow cytometry. As shown in Figure 7A, significantly more SP cells are present in the MtDP PC3 cells (~0.8%) while no apparent SP cells are detected in the WT PC3 cells. Similarly, significantly higher level of ABCG2 detection is revealed in the MtDP cells as shown in Figure 7B (~2.6% positivity in the WT vs ~5% in the MtDP cells, respectively). Considering the enhanced ALDH activity has been found in a serial of cancer stem cells [33, 34], next we evaluated ALDH activity in the WT and MtDP PC3 cells. As shown in Figure 7C, around 9 folds more ALDH^bright^ cells are found in the MtDP PC3 cells. Further, the ALDH activity of ALDH^bright^ MtDP PC3 cells is ~270% greater than the ALDH^bright^ WT PC3 cells.

We also examined the transcriptome profile on a series of common CSC markers such as *Cd44*, *Aldh1a1*, *α2β1 integrin*, *Met, Cxcr4*, *Cxcr8*, *Mmp1* and *Dpp4* (*Cd26*) [35–38]. The top three ABC family members including *Abcg2*, *Tap1* (*Abcb2*) and *Tap2* (*Abcb3*), contributing to side population feature and/or chemotherapy resistance, were also examined (Figure 7D). Other ABC-family member transcriptome profile is displayed in the Supplementary Figure S2.

## DISCUSSION

There have been reports showing that mtDNA depletion related mitochondrial dysfunction is associated with aggressive features in diverse tumors [39–43]. There is also evidence suggesting that suppressed mitochondrial function may facilitate cell stemness restoration [16, 44, 45]. In a previous study, M Higuchi et al. showed that mtDNA depletion in AR+ non-CRPC prostate cancer cells resulted in androgen independent growth [13]. Furthermore, L Moro et al. demonstrated that the CRPC PC3 cell line contained less amount of mtDNA compared to the AR+ non CRPC LNCaP cells. Taken together, it has been shown that mtDNA depletion enhances invasion capacity and anoikis resistance in LNCaP cells, the features of the AR- PC3 cell line [42]. It is also known that both invasion capacity and anoikis resistance are highly related to cancer cell stemness [46–49]. PCa, like all other carcinomas, is composed of a mixture of cells in various differentiation grades, representing different degrees of stem/progenitor cell phenotypes [50]. Thus, the use of tumor samples for correlation studies of mitochondrial function and cancer cell stemness is a challenge. To address this issue, we developed the MtDP CRPC PC3 cells, which experienced a long-term adaption process before stabilization in the culture. To avoid any deviation in using these MtDP cells, we continuously cultured the cells in the EtBr containing medium for more than 6 months, and during this period, the basic features such as cell morphology, growth rate, typical CD44 expression patterns etc., were all maintained. The cells were then used for mitochondrial function, Warburg effect and cell stemness studies.

According to previous studies, the establishment of mtDNA depletion cells was obtained by using a “dilution” strategy to eliminate mtDNA molecules, since EtBr treatment blocks mtDNA duplication [25]. However, our preliminary experiments showed that PC3 cells exhibited limited mtDNA depletion effect if such a strategy was applied to this cell line, a phenomenon not reported before. There are two issues which are unique in this cell model establishment: 1): Relative high concentration of EtBr was used in the induction process and later cell culture maintenance. As shown in Figure 1, the PC3 cells cultivated in 2μg/ml EtBr for 3 or 4 days showed no harmful effect on the cells, and the cells were actually confluent like the control cells. This may explain why there are reports about mtDNA depletion study in LNCaP, but not in PC3 cells, indicating that researchers might drop the effort in establishing mtDNA depletion cells from PC3, because most studies focus on the common concentration of EtBr, about 50ng/ml [42, 51, 52]. Another key issue in the mtDNA depletion establishment is the fact that although the cells cultivated in 2μg/ml of EtBr for 3-4 days showed no harmful on growth effect, growth inhibition happened soon after passaging, and then the cells experienced about two months adaption period, during which the cell death and cell survival gradually reached a balance. Only after this process, the cells showed stabilized slow cycling feature with the medium containing EtBr, pyruvate and uridine.

Then the question is what happened during the adaption process. We observed during the adaption experiments after 10 days that the cells started to detach from culture dish, and these cells were mostly verified dead. This might be explained by the fact that the PC3 cells in this medium were heterogeneous, and when the cells gradually lose their mtDNA content, only some cells survived through an OXPHOS independent manner. In a hierarchic composition, different cell subtypes may respond differently to mitochondrial function defects, based on their current homeostatic molecular basis especially on metabolism. Therefore, the cells survived the hit of mtDNA depletion must have metabolic reprogramming so that e.g. the extra lactate produced from the aerobic glycolysis in cytoplasm could be exported and the cytoplasmic pH homeostasis could be maintained. In contrast, the detached cells not able to adapt the mtDNA depletion-induced hit were finally dead. The cells in this adaption process or metabolic reprogramming experienced a turning point at the days 26 to 28. On about these days the cell number was the lowest, and followed by a gradual increase to the original level at day 54. It should be pointed out that the cells at day 26 and day 28 were singular and the cells gradually formed clones at about days 32 to 40, and all the cells later were derived from the colonized adapted cells.

The cells were confirmed mtDNA depleted by PCR, Western blotting and transcriptome analysis of mtDNA encoded genes. Thus, these cells must have adapted glycolysis for survival in a “forced” way. Indeed the ECAR was significantly high, and the OCR and ATP production were significantly low in these mtDNA depleted PC3 cells. It was also discovered in our experiments that the mtDNA depletion PC3 cells could consume the glucose about twice the amount of the wild type PC3 cells consumed. In line with recent studies, our transcriptome profiling of the Warburg effect related genes verified that the gene expressions of hexokinase genes *Hk2* and *Hk3*, glucose transporters *Slc2a3 (Glut3)*, *Slc2a12 (Glut12)* and *Slc2a14 (Glut14)* were all activated in the MtDP PC3 cells.

After we discovered that MtDP PC3 cells exhibited strong Warburg effect, we wanted to know whether the morphology and function of the mitochondria experienced similar reprogramming towards stem cells-like features reported previously in the human pluripotent cells [53–56]. We did find that the cells had perinuclear located mitochondria, and the transcriptome profiling of mitochondrial dynamic related genes also verified significantly higher level of fission gene *Mff* expression, and significantly lower expression levels of fusion genes including *Mfn1*, *Mfn2* and *Opa1*, which might explain mitochondrial fusion defects and mitochondrial fragmentation in these cells. Recent studies have illustrated that mitochondria dynamic balance exhibits tendentiousness towards to fission during the metabolic reprograming by cell pluripotent maintenance [53, 57]. MJ Son et al. have further demonstrated that blocking *Mfn* gene, which regulates mitochondrial fussion is favorable for pluripotency inducement [45]. But, recently L Wang et, al. also demonstrated that although mito-fission related gene *Dnm1l (Drp1)* was upregulated during inducing pluripotent cells, knocking down *Drp1* did not affect inducing pluripotent result [58]. We have observed that the mitochondrial dynamic alternations and the immature mitochondrial morphology support our hypothesis that mtDNA depletion tumor cells survive through metabolic reprogramming towards aerobic glycolysis with Warburg effect (see also Supplementary Figure S1B and S1C), hence, the survived cells may hold greater cell stemness.

To address the question whether the mtDNA depletion PC3 cells were of greater cell stemness, we examined the CD44 expression in these cells since CD44 expression has been linked to prostate cancer stem cells (CSC)s [36, 37]. It turned out that the mtDNA depletion PC3 cells expressed significantly higher level of CD44 as shown in the Figure 5A. To further explore whether the CD44 positive cells derived from this typical metabolic reprogramming, we double-stained the cells with JC-1 and fluorophore conjunct CD44 antibody. We did observe that while more than 50% of the mtDNA depletion PC3 cells were CD44^+^/Δψ_m_^Low^, only about 9.5% in the wild type PC3 had the same subtype. Instead, almost 50% of the wild type PC3 cells showed CD44^−^/Δψ_m_^High^ phenotype. It has been hypothesized that cancer stem cells have strong interlink with Warburg effect [59], and Warburg effect is associated with de-matured mitochondria [20, 56, 60]. Since our experiments have proved strong Warburg effect in the MtDP PC3 cells, the main CD44^+^/Δψ_m_^Low^ population in the MtDP PC3 cells are highly indicated to be Warburg effect and cell stemness-linked. In line with this assumption, our transcription profiling also confirmed upregulated expression of a series of other cancer stem cells related genes including *Aldh1a*, *integrin α2β1* and *Cd26* [29, 36, 61–64].

To further verify whether there is greater stemness in the mtDNA depletion PC3 cells, we next examined cell migration capability with and without hypoxia influence. It was found that the mtDNA depletion PC3 cells showed significantly greater migration capability both under normoxia and under hypoxia, and these cells even migrated more under hypoxic conditions. In contrast, the migration capability of the wild type PC3 cells was always lower, and these cells responded hypoxia with even lower migration capability as shown in Figure 5C. Although PC3 cells are aggressive in nature, only a limited proportion of the cells are verified as CSCs [65–67]. Our migration and hypoxia examinations support the previous observations, and also suggest that the mtDNA depletion PC3 cells hold greater stemness so that these cells are more prone to migrate as indicated in the previous studies [68].

Furthermore, the mtDNA depletion PC3 cells were confirmed in our study with significantly higher chemotherapeutic and radiotherapy resistance compared to the control wild type PC3 cells. Therapeutic resistance has been verified as one of the main features of CSCs, and the CSC markers ALDH activity, CD44, SP, ABCG2 etc. were considered highly associate with these common therapeutic resistant features [69–74]. In consistence with these findings, these cells were found with significantly increased SP+, ABCG2+, ALDH^bright^ and CD44+ subpopulations. In this study, we performed a modified colony formation assay for radiotherapy resistance evaluation. Instead of counting colonies where only more than 30 cells were qualified as one colony [75], we took all the single cells and cluster of cells into consideration after the irradiation treatment. We speculated that, since the mtDNA depletion cells grew slowly, and there was no enough time for these cells to grow into full clone defined by the ordinary colony assay, living cells, no matter single cells or cell clusters, all will matter for the radiotherapy resistance counting. Therefore, we decided to not plate too many cells in this assay, and each cell cluster was then explained developed from one single cell, as long as all the counted cells were confirmed viable. By such a modified colony formation assay, we discovered that the mtDNA depletion PC3 cells always showed significantly higher numbers of living cells in the groups of 5Gy and 10Gy radiotherapy, in comparison to the control cells. Collectively, all the above results show that the mtDNA depletion PC3 cells exhibit significantly greater features of CSCs in vitro. However, it should be pointed out that it is not clear in our current study whether or how if any of the mitochondrial and nucleus stress signaling interactions influence tumor cell invasion, therapeutic resistance and other cancer cell stemness-related features.

In summary, MtDP AR- CRPC PC3 cells show immature mitochondrial function with metabolic reprograming towards Warburg effect, and these cells are highly cancer stem cell-like, showing greater cell stemness including highly invasive, upregulated expression of a series of cancer stem cell markers and highly resistant to both chemotherapy and radiotherapy. Our results highlight the significance of mtDNA depletion-caused mitochondrial dysfunction in maintenance of cancer stem cells with Warburg effect. However, the current results should be explained with care due to the limitation of one cell line application in this study and the conclusion may not be universally applicable in all prostate cancers.

## MATERIALS AND METHODS

### Cell culture

Human prostate cancer cell line PC3 (ATCC Manassas, VA, USA) was routinely cultured in phenol red free RPMI-1640 medium (Gibco®) supplemented with 10% fetal bovine serum (Gibco®), 100U/ml penicillin and 100μg/ml streptomycin (Gibco®). To obtain PC3 MtDP cells, PC3 wild type cells were maintained in phenol red free RPMI-1640 medium (Gibco®) supplemented with 10% fetal bovine serum (Gibco®), 100U/ml penicillin and 100μg/ml streptomycin (Gibco®), 2μg/ml EtBr (Invitrogen™), plus 1mM sodium pyruvate and 50μg/ml uridine (Sigma Aldrich) at final concentration. After enrichment, the MtDP cells were cultured and passaged continually over 18 months to acquire a stabilized phenotype. Normoxic cultivations were performed under 37°C, 5% CO2, saturated humidity and horizontal oxygen tension (Oslo, Norway). Hypoxic cultivations were performed in an Xvivo Incubation System (Xvivo system 300C, BioSpherix) with 37°C, 5% CO2, saturated humidity and O2 concentration was set to 1.5%. An inverted phase contrast microscope was used to monitor cell status and capture images.

### PCR and transcriptome analysis

Total DNA including nuclear and mitochondrial DNA was isolated by using a PureLink® Genomic DNA Mini Kit (Invitrogen™). DNA concentration was measured by using a Nanodrop2000 UV-Vis Spectrophotometer (Thermo Fisher). Primer sequences are listed in Supplemental Experimental Procedures. Total intracellular RNA isolation was performed by using: a PureLink® RNA Mini Kit (Ambion™), homogenizer (Invitrogen), RNase Away® regent and PureLink® DNase Set (Ambion™). The total RNA samples were stored at −80°C and sent to BGI Hong Kong for analysis. Based on the BGI analysis results, differentially expressed genes between MtDP and WT PC3 cells based on FPKM (fragments per kilobase of exon per million fragments mapped) were identified.

### Immunoblotting

Cells were lysed in freshly prepared RIPA buffer supplemented with 1x Halt™ Protease/Phosphatase Inhibitor Cocktail (Thermo Scientific™). For hypoxic treated samples, the protein extraction was performed in the Xvivo Working Chamber and oxygen concentration was set to 1.5% until the lysates were centrifuged. Cleared lysates were resolved by SDS-PAGE, transferred to PVDF membrane and incubated with primary antibodies. Mouse monoclonal antibodies to MTCO1 (ab14705) and MTCO2 (ab110258) were purchased from Abcam and the monoclonal antibody for α-tubulin (T5168) was purchased from Sigma.

### Determination of cell proliferation, viability and migration

Cell proliferation curves were determined by MTT assay. The cell viabilities in chemotherapeutic treatment were determined by cell counting with trypan blue exclusion and a Countess® automatic cell counter (Life technologies). The counting results were verified by staining the samples with both Hoechst33342 and PI, a Nikon ECLIPSE E600 fluorescent microscope was used to visualize the results. The cell migration capabilities were assessed with transwell migration as detailed in Supplemental Experimental Procedures.

### Metabolic assays

Metabolic status was investigated on a XF24 Extracellular Flux analyzer (Seahorse Bioscience) with standard 24-well Seahorse microplates. A mitostress test kit was applied to evaluate the oxygen consuming ratio (OCR) and the extracellular acidification rate (ECAR). In brief, 50000 cells were seeded into plates 12~18 hours prior to analysis. Then the medium was replaced by 675μl nonbuffered Dulbecco's modified Eagle's medium containing 25mM glucose and 2mM glutamine. Cells were incubated in a CO_2_-free incubator at 37°C for 1h to allow for temperature and pH equilibration before loading into the XF24 analyzer.

Cells were seeded into 24 well plates and 500,000 cells were prepared for each well. The cells were incubated overnight to complete the attachment. After that, the medium was replaced by 400μl fresh media with or without EtBr depending on cell types, and the medium contained no cells was added into clean wells as initial controls. For each well, the glucose concentration and consumption rates were determined via a commercial kit (Sigma) based on enzymatic assays. The intracellular ATP production was assessed by using a bioluminescence assay (firefly luciferase) ATP measurement kit (Molecular Probes™) and the manufacturers' instructions were strictly followed.

### Microscopic and flow cytometric cell evaluation

Mitochondrial morphology was visualized by staining with mitochondrial specific probe MitoTracker® Red FM (Molecular Probes™) and the cell nuclei were stained by Hoechst33342 (Thermo Scientific™). The stained samples were photographed on an ECLIPSE E600 wide filed fluorescence microscope with a mercury lamp (Nikon). The bright field and dark filed microscopy was performed on a modified invert microscope. The detailed procedures are documented in Supplemental Experimental Procedures.

Flow cytometry analysis of CD44 with/without JC-1, ABCG2, side population and ALDH activities were accomplished with BD LSR II flow cytometers and a BD FACSCalibur™ flow cytometers (BD Biosciences). Details of experimental procedures are provided in Supplemental Experimental Procedures.

### Therapeutic simulation

To simulate chemotherapy, cells were treated with 0nM, 20nM and 40nM Docetaxel (Sigma) for 48h and cell viability was then assessed. To simulate radiotherapy, 1000 cells were seeded into 100mm dish and followed with an overnight incubation to allow the cells attach. Then 0Gy, 5Gy and 10Gy X-Ray irradiation were performed onto all of the dishes with an X-ray irradiator (Faxitron). More details are listed in Supplemental Experimental Procedures.

### Statistical analysis

Continuous variables are presented as mean±S.D. Statistics were conducted with a paired Student's t-test, one way or two ANOVA test (Bonferroni and LSD-t methods for further multi group comparison) depending on the features of the variables obtained. Statistical significance is represented in figures by *p<0.05, **p<0.01, ***p<0.001. All analyses were performed with the SPSS version 18 or 20 statistics software (IBM).

## SUPPLEMENTAL EXPERIMENTAL PROCEDURES


