# Correction: MtDNA depleted PC3 cells exhibit Warburg effect and cancer stem cell features

**DOI:** 10.18632/oncotarget.14800

**Published:** 2017-01-23

**Authors:** Xiaoran Li, Yali Zhong, Jie Lu, Karol Axcrona, Lars Eide, Randi G. Syljuåsen, Qian Peng, Junbai Wang, Hongquan Zhang, Mariusz Adam Goscinski, Gunnar Kvalheim, Jahn M. Nesland, Zhenhe Suo

**Present**: Due to incorrect labeling on the part of the authors, it was discovered that a different set of cell lines was used than those initially published. The authors confirmed that all the research data presented in this paper were based on in vitro studies with human CRPC DU145 cells, and not with human CRPC PC3 cells. Therefore, the correct title for this paper should be:

“MtDNA depleted DU145 cells exhibit Warburg effect and cancer stem cell features.” In addition, the gene symbols presented in this paper were mistakenly used. The correct style of gene symbols for any PCR and transcriptome analyses of DU145 and mtDNA depleted DU145 cells in this study should be with all characters italic and capitalized.

**Corrected:** The proper designation of cell lines and labels are found below. The authors deeply apologize for any confusion or inconvenience that this mistake may have caused.

Original article: Oncotarget. 2016; 7(26):40297-40313. doi: 10.18632/oncotarget.9610.

The authors regret to inform that DU145 human castration resistant prostate cancer (CRPC) cells were used instead of human CRPC PC3 cells in this paper. The authors pointed out that the mistake was due to the incorrect tube labeling, and such a mistake was discovered by a series of cell line genotyping after the publication of this manuscript. The authors confirmed that all the research data presented in this paper were based on *in vitro* studies with human CRPC DU145 cells, and not with human CRPC PC3 cells. Therefore, the correct title for this paper should be: MtDNA depleted DU145 cells exhibit Warburg effect and cancer stem cell features.

In addition, the gene symbols presented in this paper were mistakenly used, according to the guidelines for human gene nomenclature [1], in specific rule: “Names start with a lower case letter unless it is a person’s name describing a disease/ phenotype or a capitalised abbreviation e.g. AHDS “Allan-Herndon-Dudley syndrome” and ABCA1 “ATP-binding cassette, sub-family A (ABC1), member 1”, respectively ”. Therefore the correct style of gene symbols for any PCR and transcriptome analyses of DU145 and mtDNA depleted DU145 cells in this study should be with all characters italic and capitalized.

These errors do not affect any scientific measures and experimental conclusions of this paper. The authors deeply apologize for any confusion or inconvenience that this mistake may have caused. Genotyping data for the control and experimental cells are available upon request.

A complete list of specific corrections are showing as follows.

**Table:** 

#	Location	Origin	Correction	Explanations
1	In “Title”: Page 1 Linel	PC3	DU 145	Correction of cell line used
2	In “Abstract”: Page 1 Line 33	PC3	DU 145	Correction of cell line used
3	In “Abstract”: Page 1 Line 36	PC 3	DU 145	Correction of cell line used
4	In “Abstract”: Page 1 Line 37	PC 3	DU 145	Correction of cell line used
5	In “Abstract”: Page 1 Line 38	PC 3	DU 145	Correction of cell line used
6	Page 2 right column Line 19	PC 3	DU 145	Correction of cell line used
7	Page 2 right column Line 22	PC3	DU145	Correction of cell line used
8	Page 2 right column Line 25	PC3	DU 145	Correction of cell line used
9	Page 2 right column Line 30	PC 3	DU 145	Correction of cell line used
10	Page 2 right column Line 35	PC3	DU 145	Correction of cell line used
11	Page 2 right column Line 51	PC 3	DU 145	Correction of cell line used
12	Page 3 Line 2	PC 3	DU 145	Correction of cell line used
13	Page 3 Line 2	PC3	DU 145	Correction of cell line used
14	Page 3 Line 4	PC3	DU 145	Correction of cell line used
15	Page 3 Line 5	PC3	DU 145	Correction of cell line used
16	Page 3 Line 6	PC3	DU 145	Correction of cell line used
17	Page 4 left column Line 8	PC 3	DU 145	Correction of cell line used
18	Page 4 left column Line 20	PC 3	DU 145	Correction of cell line used
19	Page 4 left column Line 26	PC 3	DU 145	Correction of cell line used
20	Page 4 left column Line 30	PC 3	DU 145	Correction of cell line used
21	Page 4 left column Line 30	PC3	DU145	Correction of cell line used
22	Page 4 left column Line 35	PC3	DU 145	Correction of cell line used
23	Page 4 left column Line 39	PC 3	DU 145	Correction of cell line used
24	Page 4 left column Line 41	PC3	DU 145	Correction of cell line used
25	Page 4 left column Line 46	PC 3	DU 145	Correction of cell line used
26	Page 4 left column Line 51	PC 3	DU 145	Correction of cell line used
27	Page 4 right column Line 1	PC3	DU 145	Correction of cell line used
28	Page 4 right column Line 7	PC 3	DU 145	Correction of cell line used
29	Page 4 right column Line 8	PC3	DU 145	Correction of cell line used
30	Page 4 right column Line 18	PC3	DU145	Correction of cell line used
31	Page 4 right column Line 26	PC 3	DU 145	Correction of cell line used
32	Page 4 right column Line 30	PC 3	DU 145	Correction of cell line used
33	Page 4 right column Line 32	PC 3	DU 145	Correction of cell line used
34	Page 4 right column Line 36	PC3	DU 145	Correction of cell line used
35	Page 4 right column Line 38	PC3	DU 145	Correction of cell line used
36	Page 4 right column Line 50	PC 3	DU 145	Correction of cell line used
37	Page 4 right column Line 56	PC 3	DU 145	Correction of cell line used
38	Page 5 left column Line 2	PC 3	DU 145	Correction of cell line used
39	Page 5 left column Line 6	PC 3	DU 145	Correction of cell line used
40	Page 5 right column Line 2	PC3	DU145	Correction of cell line used
41	Page 5 bottom Line 7	PC3	DU 145	Correction of cell line used
42	Page 5 bottom Line 12	PC 3	DU 145	Correction of cell line used
43	Page 5 bottom Line 13	PC3	DU 145	Correction of cell line used
44	Page 5 bottom Line 15	PC 3	DU 145	Correction of cell line used
45	Page 6 left column Line 3	PC 3	DU 145	Correction of cell line used
46	Page 6 left column Line 4	PC3	DU 145	Correction of cell line used
47	Page 6 left column Line 5	PC3	DU 145	Correction of cell line used
48	Page 6 left column Line 5	PC3	DU 145	Correction of cell line used
49	Page 6 left column Line 7	PC3	DU 145	Correction of cell line used
50	Page 6 left column Line 9	PC 3	DU 145	Correction of cell line used
51	Page 6 left column Line 12	PC 3	DU 145	Correction of cell line used
52	Page 6 left column Line 15	PC 3	DU 145	Correction of cell line used
53	Page 6 left column Line 16	PC 3	DU 145	Correction of cell line used
54	Page 6 left column Line 18	PC3	DU145	Correction of cell line used
55	Page 6 right column Line 5	PC3	DU 145	Correction of cell line used
56	Page 6 right column Line 14	PC 3	DU 145	Correction of cell line used
57	Page 6 right column Line 14	PC3	DU 145	Correction of cell line used
58	Page 6 right column Line 19	PC 3	DU 145	Correction of cell line used
59	Page 6 bottom Line 20	PC 3	DU 145	Correction of cell line used
60	Page 6 bottom Line 20	PC3	DU 145	Correction of cell line used
61	Page 6 bottom Line 22	PC 3	DU 145	Correction of cell line used
62	Page 6 bottom Line 22	PC3	DU 145	Correction of cell line used
63	Page 6 bottom Line 23	PC3	DU145	Correction of cell line used
64	Page 7 left column Line 3	PC 3	DU 145	Correction of cell line used
65	Page 7 left column Line 6	PC 3	DU 145	Correction of cell line used
66	Page 7 right column Line 3	PC 3	DU 145	Correction of cell line used
67	Page 7 right column Line 8	PC3	DU 145	Correction of cell line used
68	Page 7 right column Line 10	PC3	DU 145	Correction of cell line used
69	Page 7 right column Line 10	PC 3	DU 145	Correction of cell line used
70	Page 7 bottom Line 11	PC 3	DU 145	Correction of cell line used
71	Page 7 bottom Line 12	PC 3	DU 145	Correction of cell line used
72	Page 7 bottom Line 12	PC 3	DU 145	Correction of cell line used
73	Page 7 bottom Line 17	PC3	DU145	Correction of cell line used
74	Page 8 left column Line 2	PC3	DU 145	Correction of cell line used
75	Page 8 left column Line 2	PC 3	DU 145	Correction of cell line used
76	Page 8 left column Line 4	PC3	DU 145	Correction of cell line used
77	Page 8 bottom Line 9	PC 3	DU 145	Correction of cell line used
78	Page 8 bottom Line 11	PC 3	DU 145	Correction of cell line used
79	Page 9 left column Line 8	PC3	DU 145	Correction of cell line used
80	Page 9 left column Line 10	PC3	DU 145	Correction of cell line used
81	Page 9 left column Line 12	PC3	DU 145	Correction of cell line used
82	Page 9 left column Line 17	PC3	DU 145	Correction of cell line used
83	Page 9 left column Line 20	PC 3	DU 145	Correction of cell line used
84	Page 9 left column Line 24	PC 3	DU 145	Correction of cell line used
85	Page 9 left column Line 34	PC 3	DU 145	Correction of cell line used
86	Page 9 left column Line 36	PC 3	DU 145	Correction of cell line used
87	Page 9 left column Line 37	PC3	DU145	Correction of cell line used
88	Page 9 left column Line 45	PC3	DU 145	Correction of cell line used
89	Page 9 left column Line 48	PC 3	DU 145	Correction of cell line used
90	Page 9 left column Line 53	PC3	DU 145	Correction of cell line used
91	Page 9 left column Line 55	PC 3	DU 145	Correction of cell line used
92	Page 9 right column Line 1	PC 3	DU 145	Correction of cell line used
93	Page 9 right column Line 8	PC3	DU 145	Correction of cell line used
94	Page 9 right column Line 9	PC 3	DU 145	Correction of cell line used
95	Page 9 right column Line 16	PC3	DU 145	Correction of cell line used
96	Page 9 right column Line 17	PC3	DU145	Correction of cell line used
97	Page 9 right column Line 18	PC 3	DU 145	Correction of cell line used
98	Page 9 right column Line 19	PC 3	DU 145	Correction of cell line used
99	Page 9 right column Line 41	PC 3	DU 145	Correction of cell line used
100	Page 9 right column Line 53	PC3	DU 145	Correction of cell line used
101	Page 10 bottom Line 1	PC3	DU 145	Correction of cell line used
102	Page 10 bottom Line 1	PC 3	DU 145	Correction of cell line used
103	Page 10 bottom Line 6	PC 3	DU 145	Correction of cell line used
104	Page 11 left column Line 11	PC 3	DU 145	Correction of cell line used
105	Page 11 right column Line 6	PC 3	DU 145	Correction of cell line used
106	Page 11 left column Line 11	PC3	DU145	Correction of cell line used
107	Page 11 bottom Line 12	PC3	DU 145	Correction of cell line used
108	Page 11 bottom Line 14	PC 3	DU 145	Correction of cell line used
109	Page 11 bottom Line 20	PC3	DU 145	Correction of cell line used
110	Page 12 left column Line 2	PC 3	DU 145	Correction of cell line used
111	Page 12 left column Line 17	PC 3	DU 145	Correction of cell line used
112	Page 12 left column Line 46	PC3	DU 145	Correction of cell line used
113	Page 120 left column Line 47	PC3	DU 145	Correction of cell line used
114	Page 20 left column Line 52	PC3	DU 145	Correction of cell line used
115	Page 12 left column Line 53	PC3	DU 145	Correction of cell line used
116	Page 12 right column Line 27	PC 3	DU 145	Correction of cell line used
117	Page 12 right column Line 36	PC 3	DU 145	Correction of cell line used
118	Page 12 right column Line 38	PC 3	DU 145	Correction of cell line used
119	Page 12 right column Line 39	PC 3	DU 145	Correction of cell line used
120	Page 12 right column Line 44	PC3	DU145	Correction of cell line used
121	Page 12 right column Line 45	PC3	DU 145	Correction of cell line used
122	Page 12 right column Line 52	PC 3	DU 145	Correction of cell line used
123	Page 12 right column Line 54	PC3	DU 145	Correction of cell line used
124	Page 13 left column Line 2	PC 3	DU 145	Correction of cell line used
125	Page 13 left column Line 4	PC 3	DU 145	Correction of cell line used
126	Page 13 left column Line 8	PC3	DU 145	Correction of cell line used
127	Page 13 left column Line 11	PC 3	DU 145	Correction of cell line used
128	Page 13 left column Line 14	PC3	DU 145	Correction of cell line used
129	Page 13 left column Line 36	PC3	DU145	Correction of cell line used
130	Page 13 left column Line 40	PC 3	DU 145	Correction of cell line used
131	Page 13 left column Line 46	PC 3	DU 145	Correction of cell line used
132	Page 13 right column Line 8	PC 3	DU 145	Correction of cell line used
133	Page 13 right column Line 8	PC3	DU 145	Correction of cell line used
134	Page 13 right column Line 12	PC3	DU 145	Correction of cell line used
135	Page 13 right column Line 13	PC3	DU 145	Correction of cell line used
136	Page 13 right column Line 44	PC3	DU 145	Correction of cell line used
137	In “Figure 2B”	PC3^WT^	DU145^WT^	Correction of cell line used
138	In “Figure 2B”	pc3^MtDP^	DU145^MtDP^	Correction of cell line used
139	In “Figure 3B”	PC3^WT^	DU145^WT^	Correction of cell line used
140	In “Figure 3B”	PC3^MtDP^	DU145^MtDP^	Correction of cell line used
141	In “Figure 4D”	PC3^WT^	DU145^WT^	Correction of cell line used
142	In “Figure 4D”	pc3^MtDP^	DU145^MtDP^	Correction of cell line used
143	In “Figure 5C”	PC3	DU 145	Correction of cell line used
144	In “Figure 5C”	PC3	DU145	Correction of cell line used
145	In “Figure 7D”	PC3^WT^	DU145^WT^	Correction of cell line used
146	In “Figure 7D”	PC3^MtDP^	DU145^MtDP^	Correction of cell line used

**For Supplementary Material**				
147	Page 1 left column Line 34	PC3	DU 145	Correction of cell line used
148	Page 1 left column Line 34	PC3	DU 145	Correction of cell line used
149	Page 3 bottom Line 1	PC3	DU 145	Correction of cell line used
150	Page 4 left column Line 1	PC3	DU 145	Correction of cell line used
151	In “Supplementary Figure SI A”	PC3	DU 145	Correction of cell line used
152	In “Supplementary Figure SIB”	PC3	DU 145	Correction of cell line used
153	In “Supplementary Figure SIC”	PC3	DU 145	Correction of cell line used
154	In “Supplementary Figure S2”	PC3^WT^	DU145^WT^	Correction of cell line used
155	In “Supplementary Figure S2”	pC3^MtDP^	DU145^MtDP^	Correction of cell line used
