# Supplementary material for: MtDNA depleted PC3 cells exhibit Warburg effect and cancer stem cell features
Source: Oncotarget. 2016 May 26;7(26):40297–313. doi: 10.18632/oncotarget.9610 (PMC5130009; doi:10.18632/oncotarget.9610)
Supplement: Supplementary file 1 [file oncotarget-07-40297-s001.pdf]

MtDNA depleted PC3 cells exhibit Warburg effect and cancer stem cell features

SUPPLEMENTAL EXPERIMENTAL PROCEDURES

PCR analysis

Primer sequences were as follows: 5′-ACAGCCACTTTCCACA-3′ sense and 5′-CTGGTGT TAGGGTTCTT-3′ antisense for D-loop, 5′-CCTACT TACAGGGCTCAA-3′ sense and 5′-GAGGGATAGG AGGAGAAT-3′ antisense for Nd4, 5′-CCTCAAGATCA TCAGCAATGC-3′ sense and 5′-TGGTCATGAGTC CTTCCACG-3′ antisense for nuclear DNA encoded glyceraldehyde 3-phosphate dehydrogenase (Gapdh). Annealing temperatures presented above were used as follows: 52°C for D-loop, 49 °C for Nd4, 58°C for Gapdh.

Transwell migration assay

To investigate cell mobilization, the 8µm pore polycarbonate membrane inserts (transwell® Corning) were applied. For each group, 6000 cells were loaded into insert chambers with RPMI1640 medium FBS free. The low well was filled with 500µl RPMI1640 medium which containing 10% FBS and 200ng/ml G-CSF. After 24 hours incubation, the insert chambers were fixed with methanol and stained with 0.1% crystal violet in methanol. After that the unpenetrated cells were removed by cotton swab and the polycarbonate membranes were dried under room temperature. The membranes were cut and mounted on slides before absolute penetrated cells were counted.

Mitochondrial morphology assessment

PC3 WT and PC3 MtDP cells were seeded in glass-bottom culture dishes with respective culture medium for 48 hours normal incubation. To visualize mitochondria, the culture medium were replaced with FluoroBrite™ DMEM (Gibco®) contains 50nM MitoTracker® Red FM (Molecular Probes™) and 10% FBS for 30 min incubation at 37°C under 5% CO2. Before microscopic observation, the medium were replaced with FluoroBrite™ DMEM contains 2µg/ml Hoechst33342 and proceeded on incubation for 10 min. After that, cells were washed with cold PBS, immediately the fluorochromes were visualized on a Nikon ECLIPSE E600 fluorescence microscope, and the observations were photographed with a customized optical adapter and digital camera (Canon).

Mitochondrial membrane potential and CD44 co-analysis

Mitochondrial membrane potential probe JC-1 (Molecular Probes™) was dissolved in DMSO (Sigma) and prepared into 2mM stock solution. Staining medium was prepared by FluoroBrite™ DMEM with 1µM JC-1 and 10% FBS. The main staining protocol was referenced to the manufacturer's suggestion. Cells treated with CCCP were applied as controls. For the CD44 expression co-analysis, cells were harvested by TrypLE™ Express (Gibco®) and blocked with 0.5% goat serum in FluoroBrite™ DMEM for 15 min prior to the antibody staining. Then the cells were spined and resuspended in different staining media, for which antibody and isotype control antibody were added, and at meantime an unstained sample was prepared as control. To avoid excitation spectral overlap, we chose an allophycocyanin (APC) labeled monoclonal anti-CD44 antibody (BD Pharmingen) to perform the co-analysis with JC-1 based mitochondrial membrane potential staining. The stain procedure was strictly stuck to the instructions and the cells stained with isotype control antibody were utilized to set positive gate. PI was added just before test in order to exclude dead cells. Data were acquired on a customized BD LSR II flowcytometer (BD Biosciences), 355nm UV laser was utilized to excite PI (610/20 filter set), 488nm laser was utilized to excite JC-1 (530/30 filter set for monomer and 610/20 filter set for aggregates) and 25% compensation was applied to separate signal (JC-1 green – JC-1 red); 633nm laser was utilized to collected APC signal via filter set 660/20. Data were analyzed and presented with Flowjo v7.6.

Side population assay

Cells were cultured up to 60% confluence in 100mm dish and then were harvested with TrypLE™ Express regent. Approximated $1*10^6$ cells were collected and maintained in prewarmed RPMI1640 medium (phenol red free), which contained 2% fetal bovine serum. Hoechst33342 stock solution (1mg/ml) was then added to a final concentration of 5µg/ml and mixed well, and the cell suspensions were incubated with intermittent shaking for 90 min in 37°C water bath and avoided from light. For controls, cells treated with 50µM verapamil

were stained with Hoechst33342 as described above. After the incubation, the cells were washed with ice-cold HBSS+ solution (HBSS buffer containing 2% FBS) and centrifuged at 4°C. The cells were re-suspended in ice-cold HBSS+ and placed on ice until examined on flowcytometer. Before test, cells were filtered through a 70μm cell strainer and 1μl 1mg/ml PI were added to exclude dead cells. Cells were examined on a customized BD LSRII flow cytometer. Hoechst33342 dye was excited with a UV laser at 350nm, Hoechst blue signal was detected with 450/50 filter set and Hoechst red signal was detected with 610/20 filter set. The procedure referred to Goodell et al. has been previously published [1].

Flow cytometry assessment of ABCG2, and CD44

Cells were treated with TrypLE™ Express regent and prepared into cell suspensions, and then $1*10^6$ cells were collected into 5ml tubes. The cells were then spun, washed with cold PBS and re-suspended in FluoroBrite™ DMEM medium containing 0.5% goat serum or IgG free BSA depend on the antibody instruction. After that, cells were incubated in 37°C water bath for 15 min pre-blocking, and the cells were stained by antibodies directly conjugated with fluorophores. For each sample, an unstained sample and isotype control antibody stained sample were prepared for gating. The antibodies were all purchased from BD Pharmingen: monoclonal anti CD44 antibody conjugated with appophycoyanin (APC), and monoclonal anti-CD338 (ABCG2) antibody conjugated with APC (included the fluorophores labeled isotype control antibodies, respectively). The antibodies were added into pre-blocked cell suspensions, mixed well and incubated at 37°C without light exposure for 25min. Cell suspensions were washed twice with PBS and re-suspended in HBSS+ buffer. Before test, cells were filtered through a 70μm cell strainer and 1μl 1mg/ml PI was added to exclude dead cells. Samples were analyzed on a BD LSRII flowcytometer.

ALDH activity analysis

To measure ALDH activity, a commercial ALDEFLUOR™ kit was obtained from STEMCELL™ TECHNOLOGIES Company for flow cytometry test. The preparation of related regents and general staining procedure were strictly referred to the instruction. Cells around 70% confluence were dissociated with TrypLE™ Express regent (Thermo Fisher) and the cell number was counted. Then cells were suspended in ALDEFLUOR™ assay buffer containing activated ALDEFLUOR™ regent, 5μl for $1*10^6$ cells in 1ml buffer. For staining control, fresh prepared 500μl cell staining sample were transferred into a new tube contains 5μl ALDH inhibitor (DEAB, STEMCELL™ TECHNOLOGIES). All samples were generally mixed well and maintained in a 37°C water bath without light exposure for 30 min. Aldefluor fluorescence was excited by 488 nm laser, and the fluorescence was detected using 530/30 nm band pass filter set by a BD LSR II flowcytometer as previous described. The cells were stained with sufficient PI before analyzing, and the dead cells were excluded from gating. Software Flowjo version 10 was applied for further data analyze and present.

Radiotherapy

1000 cells were seeded into 100mm dish and followed with an overnight incubation to allow the cells attach. Then 0Gy, 5Gy and 10Gy X-Ray irradiations were performed onto all of the dishes. For the first four days, the medium was kept unchanged, after that the medium was changed for every other day. Around 15 days' cultivation visible colons were formed, and the cells were fixed with 4% paraformaldehyde (PFA) and visualized by staining with 0.1% (w/v) crystal violet in methanol. Then the dishes were washed and dried before the colonies were counted in a G: BOX F3 multi-function imaging system with related software (Syngene). The microscopic counting and photograph were carried out on a customized dark-field microscope. For 5Gy and 10Gy treatments, isolated single cells and colonies constructed by over 30 cells were counted as potential colonies and a colony.

REFERENCES

1. Goodell MA. Stem cell identification and sorting using the Hoechst 33342 side population (SP). Current protocols in cytometry / editorial board, J Paul Robinson, managing editor [et al]. 2005; Chapter 9:Unit9 18.

SUPPLEMENTARY FIGURES

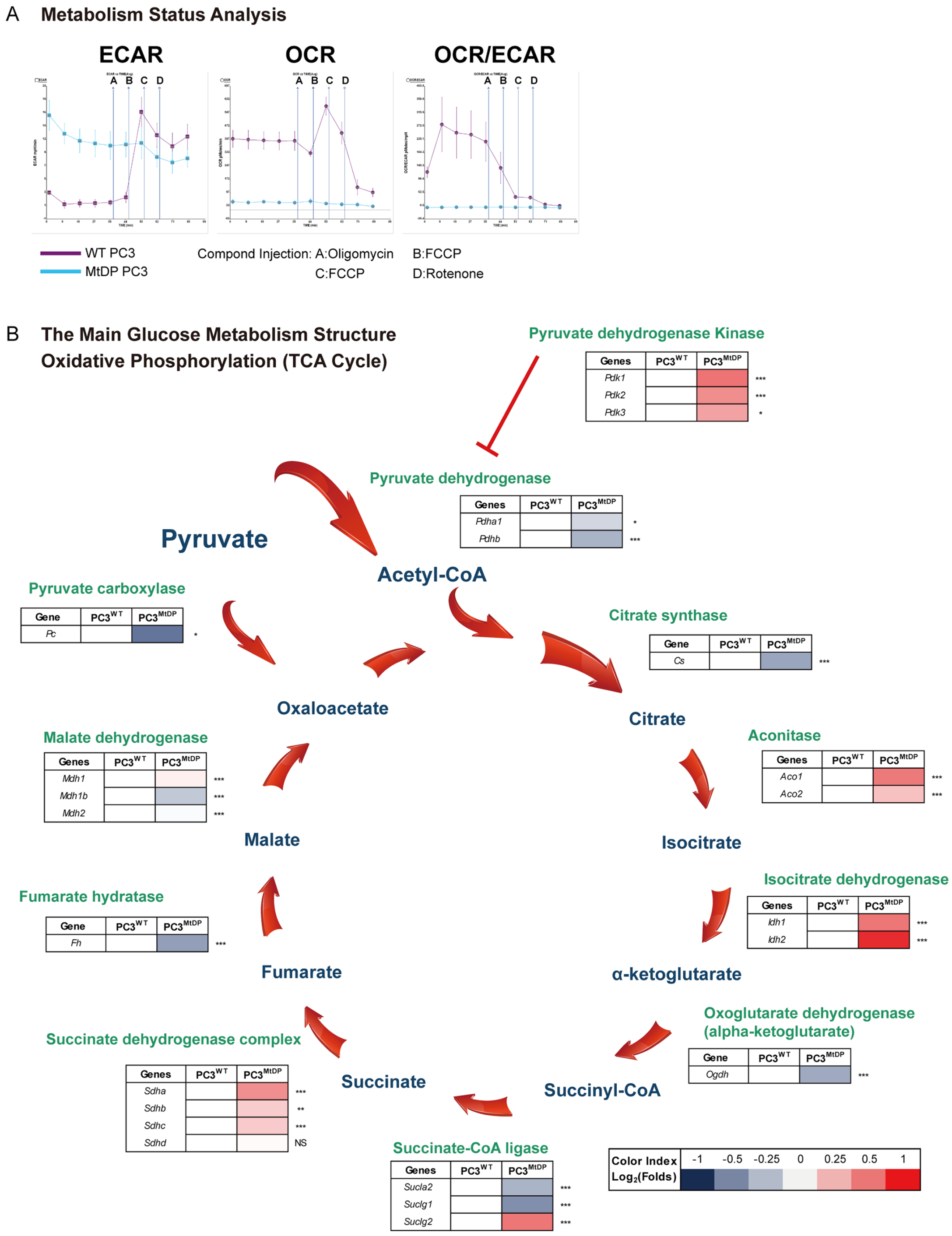

Supplementary Figure S1: A. Mitochondrial functions were evaluated with a modified mitostress assay on a XF24 Extracellular Flux analyzer (Seahorse Bioscience). Represent results including ECAR, OCR and OCR/ECAR ratio values are shown with line charts. Three experiments were carried out and the data are represented as means ± SD. **B.** The reprogrammed main glucose metabolism pathways in MtDP PC3 cells. The heat maps represent transcriptome analysis of glycolysis and OXPHOS related genes. The ratio is indicated by a color-coded index bar. (FPKM value, Log_2 (MtDP/WT)). Statistical significance: *p<0.05, **p<0.01, ***p<0.001. (*Continued*)

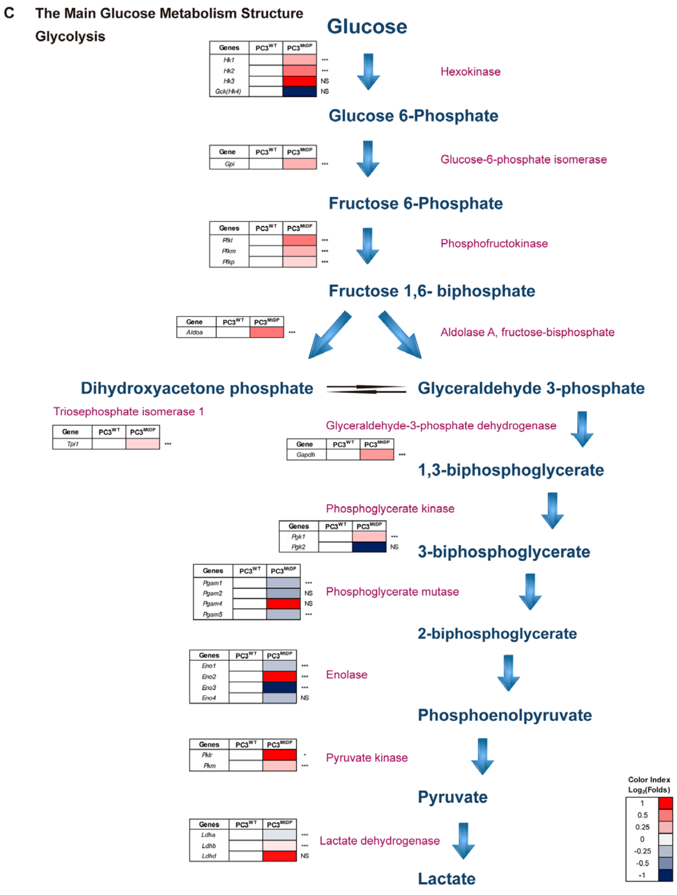

Supplementary Figure S1: (*Continued*) C. The reprogrammed main glucose metabolism pathways in MtDP PC3 cells. The heat maps represent transcriptome analysis of glycolysis and OXPHOS related genes. The ratio is indicated by a color-coded index bar. (FPKM value, Log_2 (MtDP/WT)). Statistical significance: *p<0.05, **p<0.01, ***p<0.001.

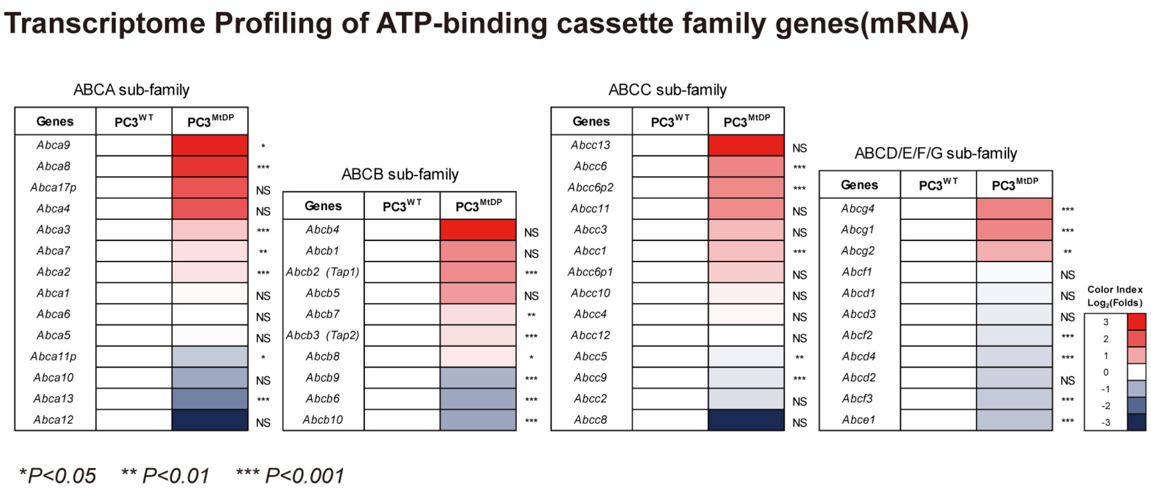

Supplementary Figure S2: Transcriptome analysis of ABC-family genes in WT and MtDP PC3 cells. The ratio is indicated by a color-coded index bar. (FPKM value, Log_2 (MtDP/WT)). Statistical significance: *p<0.05, **p<0.01, ***p<0.001.
